# Serum Activin A Level and 1‐Year Mortality in Atrial Septal Defect‐Associated Pulmonary Artery Hypertension: A Case‐Control Study From the COHARD‐PH Registry

**DOI:** 10.1155/ijvm/7787559

**Published:** 2026-05-25

**Authors:** Pranindya Rinastiti, Anggoro Budi Hartopo, Cindy Elica Cipta, Syallom Gita Maharani, Dyah Wulan Anggrahini, Noriaki Emoto, Lucia Kris Dinarti

**Affiliations:** ^1^ Department of Cardiology and Vascular Medicine, Faculty of Medicine, Public Health and Nursing Universitas Gadjah Mada-Dr. Sardjito Hospital, Yogyakarta, Indonesia, ugm.ac.id; ^2^ Laboratory of Clinical Pharmaceutical Science, Kobe Pharmaceutical University, Kobe, Japan, kobepharma-u.ac.jp; ^3^ Division of Cardiovascular Medicine, Department of Internal Medicine, Kobe University Graduate School of Medicine, Kobe, Japan, kobe-u.ac.jp

## Abstract

**Background:**

Serum activin A level is a promising prognostic biomarker for pulmonary artery hypertension (PAH). In this study, we aim to investigate serum activin A levels and their associations with 1‐year mortality in atrial septal defect (ASD)–associated PAH patients.

**Methods:**

This was a case‐control study of adult Indonesian patients diagnosed with ASD‐PAH from the COHARD‐PH registry. Cases were subjects deceased in a 1‐year follow‐up, whereas controls were those survived. Serum activin A was measured at the index of diagnosis. The demographics, clinical parameters, hemodynamic, and mortality data were retrieved from the registry database up to a 1‐year follow‐up period. These data were compared and analyzed between the case and control groups.

**Results:**

From the 1‐year follow‐up data, 44 cases (deceased group) and 102 controls (survived group) were identified. The deceased group had significant lower bodyweight (*p* < 0.001), body mass index (BMI) (*p* < 0.01), with higher NT‐proBNP level (< 0.0001), higher proportion of Eisenmenger syndrome (*p* < 0.05), higher right atrial (RA) area (*p* < 0.0001), lower tricuspid annular plane systolic excursion (TAPSE) (*p* < 0.01), and a significant increase in mean right atrial pressure (mRAP) (< 0.0001). There was no difference in the activin A level between deceased and survivors (506.3 ± 259.1 pg/mL vs. 536.1 ± 293.9 pg/mL, *p* > 0.05, respectively). Moreover, activin A level did not associate with the mortality risk among subjects (OR = 0.9996; 95% CI: 0.9982–1.00087; *p* > 0.05).

**Conclusion:**

Serum activin A level did not associate with 1‐year mortality in adult patients with ASD‐associated PAH.

## 1. Introduction

Pulmonary vascular remodeling is the pathophysiology hallmark of pulmonary artery hypertension (PAH). Current studies have discovered the important role of transforming growth factor beta (TGF‐*β*) superfamily and bone morphogenetic protein receptor type II (BMPRII) dysregulated signaling in the development of pulmonary vascular remodeling [1]. At the molecular level, studies showed that an imbalance of TGF‐*β* superfamily signaling resulted in a significant upregulation of activin A, a glycoprotein from the TGF‐*β* superfamily, in pulmonary vasculatures that aggravated the remodeling process [1, 2]. Activin A, through activin receptors (ACTRIIB and ACTRIIA), reduces BMPRII levels in pulmonary endothelial cells and its downstream Smad1/5‐pathway that leads to the progression of obstructive vascular remodeling in PAH [3].

Clinical studies confirmed increased serum activin A levels in patients with PAH, and its higher level could act as a predictor for clinical adverse outcomes [4–6]. However, all these studies did not include congenital heart disease (CHD)–associated PAH, a subtype of Group 1 pulmonary hypertension (PH) that is still prevalent in developing countries [7]. A previous study from our registry indicated that serum activin A levels were increased in CHD‐associated PAH, and it serves as a promising biomarker to discriminate CHD‐associated PAH from CHD without PAH [8]. In this current study, we aim to investigate serum activin A levels and their associations with 1‐year mortality, specifically among atrial septal defect (ASD)–associated PAH patients.

## 2. Material and Methods

### 2.1. Study Design, Setting, and Participants

This study employed a case‐control method from a retrospective hospital‐based registry database. The cases consisted of subjects who had deceased, and the controls consisted of subjects who survived. The subjects were adult Indonesian patients diagnosed with CHD‐associated PAH derived from the COngenital HeARt Disease in the adult and Pulmonary Hypertension (COHARD‐PH) registry. The COHARD‐PH registry is a single‐center hospital‐based registry collecting adult patients with CHD and PH presented in Dr. Sardjito Hospital, Yogyakarta, Indonesia [9]. Because most patients in the COHARD‐PH registry had defects on interatrial septum or ASD, we selected subjects with ASD (*n* = 1370) registered from 2012 to 2023 registry with inclusion criteria as follows: (1) patients diagnosed with ASD, (2) patients with PH or PAH after right heart catheterization (RHC) procedure, (3) patients with frozen blood samples sufficient for activin A measurements, and (4) patients with at least 1‐year follow‐up data after the index of diagnosis. The exclusion criteria were (1) out‐of‐range measurements of activin A level by the ELISA method and (2) missing important variable data. Based on the inclusion criteria, 671 patients were eligible to become subjects of this study. The Medical and Health Research Ethics Committee of the Faculty of Medicine, Public Health and Nursing, Universitas Gadjah Mada, and Dr. Sardjito Hospital, Yogyakarta, approved the study (Number KE/FK/1697/EC/2024).

### 2.2. Demography, Clinical, and Hemodynamic Data Measurement

The demography, clinical, and hemodynamic data were retrieved from the COHARD‐PH registry database. Clinical data were bodyweight, body mass index (BMI), systolic blood pressure, heart rate, WHO‐functional class, and 6‐min walking distance (6MWD). Systolic blood pressure and heart rate were measured from the upper extremity with a calibrated digital tensimeter during outpatient visits at the index of PAH diagnosis. A 6MWD was derived from a 6‐min walking test (6MWT) conducted by trained nurses. Eisenmenger syndrome was defined as patients with large ASD and dominant right‐to‐left shunts, central cyanosis, secondary polycythemia, and chronic hypoxemia.

Transthoracic echocardiography (TTE), transesophageal echocardiography (TOE), and RHC were performed following standard diagnostic protocols as previously outlined [9]. Echocardiographic parameters assessed included the right atrial (RA) area, estimated RA pressure, tricuspid annular plane systolic excursion (TAPSE), tricuspid valve gradient (TVG), tricuspid valve regurgitation, and left ventricular ejection fraction (LVEF), all following established procedures. Pulmonary artery hypertension (PAH) was diagnosed based on RHC findings, with measurements including mean pulmonary artery pressure (mPAP), mean right atrial pressure (mRAP), pulmonary vascular resistance index (PVRi), and flow ratio obtained in accordance with established recommendations [7]. The index of PAH diagnosis was determined by RHC.

### 2.3. Laboratory Tests

Blood samples for activin A examination were withdrawn from the peripheral veins at the index diagnosis of PAH, namely, during the RHC procedure. Blood examination for hemoglobin, hematocrit, and creatinine level was performed in the hospital laboratory, using standard equipment (Sysmex, Kobe, Japan). For NT‐proBNP measurement, similar blood samples during RHC were applied to the electrochemiluminescence immunoassay (Elecsys ProBNP II) and a Cobas e immunoassay analyzer (Roche Diagnostics, Germany). NT‐proBNP measurement was performed in the hospital laboratory. For biomarker measurement, blood samples were withdrawn from peripheral veins during the RHC procedure and centrifuged at 2000 × g for 10 min at room temperature. The supernatant was collected to obtain serum, which was subsequently aliquoted and frozen at −80°C freezer in the Biobank Unit, Faculty of Medicine, Public Health, and Nursing Universitas Gadjah Mada, until assayed. Activin A levels were measured using the Human/Mouse/Rat Activin A Quantikine ELISA Kit (DAC00B, R&D Systems, Minneapolis, Minnesota, United States), according to the manufacturer’s protocol. The assay was conducted in the Integrated Research Laboratory (*Laboratorium Riset Terpadu*), Faculty of Medicine, Public Health and Nursing Universitas Gadjah Mada. The aliquoted frozen sera were thawed once and applied to the ELISA kit unduplicated. The signal of ELISA was read on 450 nm nanodrop [8].

### 2.4. Outcome Measurement: 1‐Year Mortality

The outcome was a 1‐year mortality. Research assistants regularly followed up each subject via phone to gather information on the major events, including hospitalization and survival, and stored the data in the COHARD‐PH registry database. Most subjects regularly visited the outpatient clinic of our hospital. Those who did not perform the regular visit were contacted by phone. The survival data were collected based on the last follow‐up call to each subject, within 1 year from the index of PAH diagnosis.

### 2.5. Statistical Analysis

For statistical analysis, continuous variables were expressed as mean ± standard deviation (SD) with normality assessed using the Shapiro–Wilk or Kolmogorov–Smirnov tests. Differences among groups were tested using Student’s *t*‐test for normally distributed continuous data or the Mann–Whitney test for nonparametric data. Categorical variables were compared using the chi‐square test. Correlations between continuous variables were evaluated using Pearson’s correlation. Logistic regression analysis was used to identify activin A as a predictor of mortality. To analyze the prognostic values, we employed ROC analysis to determine the cut‐off value. The Kaplan–Meier analysis was performed based on the cut‐off value starting from the baseline visit to a 1‐year follow‐up period. Statistical significance was defined as *p* value < 0.05. All analyses were conducted in GraphPad Prism Version 8.0.1 (GraphPad Software, La Jolla, California, United States).

## 3. Results

**Figure 1:**
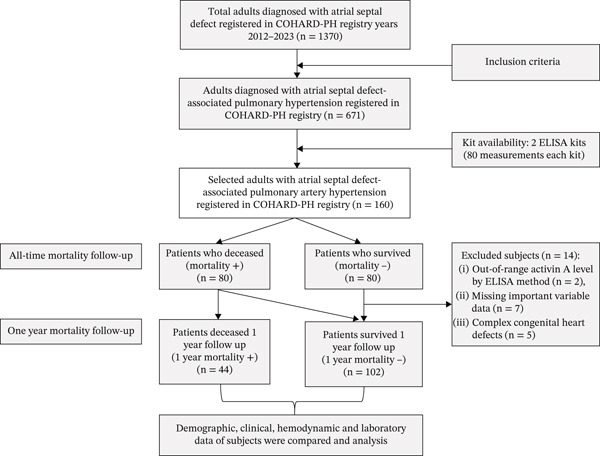
Flowchart of patient inclusion for this study.

From the COHARD‐PH registry, 671 patients with ASD‐associated PH or PAH were eligible for the study. Due to budget constraints, we could not measure activin A levels in all those patients. Therefore, as many as 160 patients with ASD‐associated PAH were selected as subjects, whose blood samples were sufficient for 2 ELISA kit measurements (@ 80 measurements per kit), consisted of 80 deceased subjects (as cases) and 80 survived subjects (as controls). Both deceased and survived subjects were selected consecutively, based on the date of their inclusion in the COHARD‐PH registry. The baseline characteristics of all patients (*n* = 671) compared to selected patients (*n* = 160) and nonselected patients (*n* = 511) are depicted in Table S1. Of 160 subjects, 14 subjects were excluded due to out‐of‐range activin A measurement (nondetected level) (*n* = 2), missing important variable data (*n* = 7), and subjects who were later diagnosed as complex congenital heart defects (*n* = 5). A total of 146 subjects were analyzed in this study. Later, we selected subjects who had deceased within a 1‐year follow‐up (cases, *n* = 44) and subjects who survived (unmatched controls, *n* = 102). The flowchart of subjects’ selection is depicted in Figure 1. The deceased subjects had PH‐related death, either died during hospitalization due to PH crises or right heart failure or out‐of‐hospital death. Deceased subjects who suffered out‐of‐hospital death were investigated by verbal autopsy to determine the cause of death, and all were pronounced PH‐related deaths. Table 1 shows the characteristics of subjects at the index of PAH diagnosis and their mortality status at 1‐year follow‐up.

**Table 1:** Clinical, echocardiography, and hemodynamic characteristics of all subjects at the index of PAH diagnosis and their status at 1‐year follow‐up.

Characteristics	All subjects (*n* = 146)	Cases (deceased) (*n* = 44)	Controls (survived) (*n* = 102)	*p* value
Clinical characteristics (mean ± SD)				
Females sex, *n* (%)	119 (81.5)	37 (84.1)	82 (80.4)	0.597
Age (years)	37.63 ± 13.24	35.68 ± 11.62	38.47 ± 13.85	0.327
Bodyweight (kg) (*n* = 143)	46.85 ± 12.11	41.79 ± 10.37	48.96 ± 12.2	0.0004
BMI (kg/m^2^) (*n* = 144)	18.95 ± 4.72	17.39 ± 3.38	19.79 ± 4.68	0.004
SBP (mmHg) (*n* = 131)	133.2 ± 23.05	128.2 ± 20.42	134.8 ± 23.69	0.145
HR (beat/min) (*n* = 131)	85.74 ± 16.89	87.26 ± 20.01	85.27 ± 15.89	0.997
Hemoglobin (g/dL) (*n* = 139)	14.25 ± 2.151	14.25 ± 2.73	14.25 ± 1.91	0.455
Hematocrit (%) (*n* = 138)	43.14 ± 6.55	43.60 ± 8.40	42.98 ± 5.80	0.575
Creatinine (g/dL) (*n* = 137)	0.89 ± 0.39	0.99 ± 0.66	0.86 ± 0.23	0.396
6MWD (m) (*n* = 101)	283.20 ± 94.69	267.30 ± 111.20	288.80 ± 88.43	0.518
WHO f.c., *n* (%) (*n* = 130)				
I–II	101 (77.7)	27 (69.2)	74 (81.3)	0.130
III–IV	29 (22.3)	12 (30.8)	17 (18.7)	
Eisenmenger syndrome, *n* (%) (*n* = 144)	27 (18.8)	13 (30.2)	14 (14.0)	0.023
Echocardiography characteristics (mean ± SD)				
RA area (cm^2^) (*n* = 110)	36.69 ± 14.85	45.24 ± 13.76	33.27 ± 13.91	< 0.0001
RA pressure (mmHg) (*n* = 141)	6.70 ± 4.52	7.84 ± 5.37	6.20 ± 4.03	0.180
TAPSE (mm) (*n* = 146)	22.48 ± 5.69	20.52 ± 5.64	23.32 ± 5.52	0.0027
TVG (mmHg) (*n* = 144)	75.42 ± 32.86	81.4 ± 31.93	72.87 ± 33.08	0.139
LVEF (%) (*n* = 146)	70.59 ± 8.49	69.02 ± 8.85	71.26 ± 8.29	0.271
Pericardial effusion (*n* = 142), *n* (%)	14 (9.8)	5 (3.5)	9 (6.3)	0.687
Hemodynamics characteristics (mean ± SD)				
mPAP (mmHg) (*n* = 129)	49.1 ± 17.77	47.74 ± 15.39	49.46 ± 18.4	0.686
mRAP (mmHg) (*n* = 128)	10.38 ± 5.563	14.64 ± 6.17	9.18 ± 4.76	< 0.0001
PVRi (WU.m^2^)	14.12 ± 13.61	14.88 ± 18.11	13.94 ± 12.43	0.506
Flow ratio	2.15 ± 1.38	2.41 ± 1.65	2.09 ± 1.30	0.489
Biomarkers (mean ± SD)				
NT‐proBNP (pg/mL) (*n* = 110)	3,628.0 ± 5,845.0	8,287.0 ± 9,629.0	2,037.0 ± 2,235.0	< 0.0001
Activin A (pg/mL)	527.1 ± 283.3	506.3 ± 259.1	536.1 ± 293.9	0.558
PAH‐specific treatment (*n* [%])				
Sildenafil	70 (47.9)	21 (47.7)	49 (48.0)	0.641
Sildenafil + beraprost	20 (13.7)	9 (20.5)	11 (10.8)	

Abbreviations: 6MWD, 6‐min walking distance; BMI, body mass index; HR, heart rate; LVEF, left ventricular ejection fraction; mPAP, mean pulmonary artery pressure; mRAP, mean right atrial pressure; NT‐proBNP, N terminal pro B type natriuretic peptide; PVRi, pulmonary vascular resistance index; RA, right atrium; SBP, systolic blood pressure; SD, standard deviation; TAPSE, tricuspid annular plane systolic excursion; TVG, tricuspid valve gradient; WHO f.c., World Health Organization functional class; WU, Wood unit.

The demographic characteristics showed a mean age of 37.63 ± 13.24 years, with the majority of the subjects being females (81.5%). There were almost no significant differences in the demographic characteristics between cases (deceased subjects) and controls (survived subjects). However, in the cases, subjects had significant lower bodyweight (48.96 ± 12.2 vs. 41.79 ± 10.37 kg, *p* < 0.0005), BMI (19.79 ± 4.68 vs. 17.39 ± 3.38 kg/m^2^, *p* < 0.005), with higher NT‐proBNP level (2,037.0 ± 2,235.0 vs. 8,287.0 ± 9,629.0 pg/mL, *p* < 0.0001), and higher proportion of Eisenmenger syndrome (14% vs. 30.2%, *p* < 0.05). Echocardiography data also showed higher RA area (33.27 ± 13.91 vs. 45.24 ± 13.76 cm^2^, *p* < 0.0001) and lower TAPSE (23.32 ± 5.52 vs. 20.52 ± 5.64 mm, *p* < 0.005), consistent with a significant increase in mRAP (9.18 ± 4.76 vs. 14.64 ± 6.17 mmHg, < 0.0001) obtained during the RHC procedure. PAH‐specific medication available in Indonesia was sildenafil and beraprost, which were given to 90 subjects (61.6%) in a similar proportion between groups.

Continuous correlation analysis of activin A also revealed that this biomarker has a significant positive correlation with age, systolic blood pressure, and RA pressure, while RA area showed a negative correlation with activin A level (Table 2).

**Table 2:** Correlation test between serum activin A level and continuous variables.

Parameters	Correlation	
	*r*value	*p*value
Age (years)	0.316	< 0.0001
Bodyweight (kg)	0.088	0.295
BMI (kg/m^2^)	0.119	0.154
SBP (mmHg)	0.254	0.003
HR (beat/min)	−0.001	0.994
Hemoglobin (g/dL)	−0.018	0.836
Hematocrit (%)	−0.01	0.9
Creatinine (g/dL)	0.042	0.624
6MWD (m)	0.114	0.258
NT‐proBNP (pg/mL)	0.017	0.858
RA area (cm^2^)	−0.18	0.033
RA pressure (mmHg)	0.165	0.05
TVG (mmHg)	0.012	0.883
TAPSE (mm)	0.02	0.814
LVEF (%)	−0.028	0.738
mPAP (mmHg)	0.042	0.634
mRAP (mmHg)	0.064	0.474
PVRi (WU.m^2^)	0.119	0.183
Flow ratio	−0.156	0.079

Abbreviations: 6MWD, 6‐min walking distance; BMI, body mass index; HR, heart rate; LVEF, left ventricular ejection fraction; mPAP, mean pulmonary artery pressure; mRAP, mean right atrial pressure; PNT‐proBNP, N terminal pro B type natriuretic peptide; RA, right atrial; SBP, systolic blood pressure; TAPSE, tricuspid annular plane systolic excursion; TVG, tricuspid valve gradient; PVRI, pulmonary vascular resistance index; WU, Wood unit.

**Figure 2:**
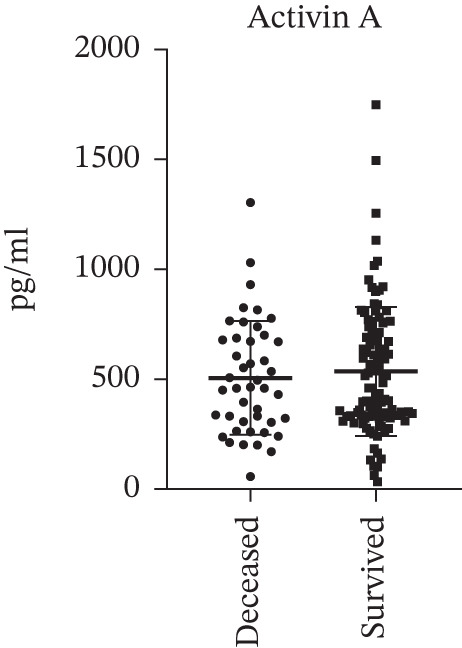
Baseline activin A level did not significantly differ between cases (deceased subjects) and controls (survived subjects) at 1‐year follow‐up from the index of PAH diagnosis (mean ± SD: 506.3 ± 259.1 pg/mL vs. 536.1 ± 293.9 pg/mL, *p* > 0.05, respectively).

Despite the change in the echocardiography and hemodynamic parameters, as well as significant correlation with some echocardiography parameters, there was no difference in the activin A level between cases (deceased) and controls (survived subjects) at 1‐year follow‐up (506.3 ± 259.1 pg/mL vs. 536.1 ± 293.9 pg/mL, *p* > 0.05, respectively) (Figure 2 and Table 3). The level of activin A also showed no correlation with the 1‐year mortality among the subjects (*r* = −0.049, *p* > 0.05). From logistic regression analysis, the level of activin A did not associate with the mortality risk among the subjects (OR = 0.9996; 95% CI: 0.9982–1.00087; *p* > 0.05) (Table 4).

**Table 3:** Baseline activin A level between cases (deceased) and controls (survived) and its correlation to 1‐year mortality.

Variables	Cases (deceased) (*n* = 44)	Controls (survived) (*n* = 102)	*p* value	Correlation	
				*R*	*p* value
Activin A (pg/mL), mean ± SD	506.3 ± 259.1	536.1 ± 293.9	0.558	−0.049	0.562

**Table 4:** Risk factor of mortality (odds ratio [OR] of mortality estimated by logistic regression model).

	Unadjusted OR	95% CI	*R* ^2^	*p* value
Activin A (pg/mL)	0.9996	0.9982–1.000087	0.0022	0.565

**Figure 3:**
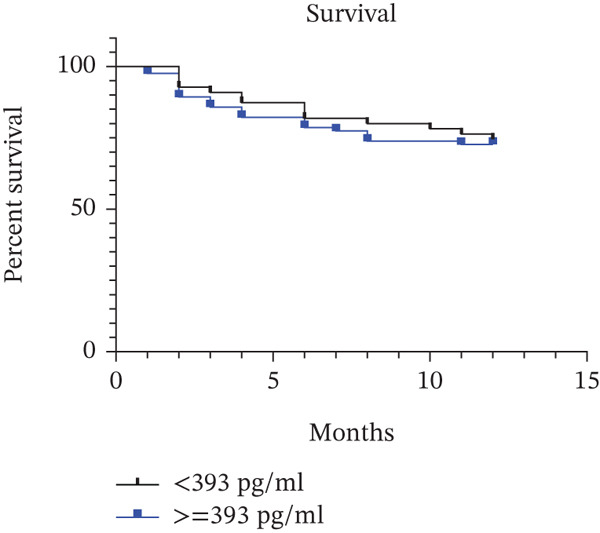
Kaplan–Meier for survival curves according to levels of activin A (cut‐off value 393 pg/mL).

We have performed receiver operating characteristic (ROC) analysis with our data, but it yielded a low predictive discriminant for 1‐year mortality. To validate the prognostic value of activin A, we used the previously established cut‐off value. A previous cohort in PAH suggested a prognostic cut‐off value for activin A at baseline [6]. However, our result shows that subjects with an activin A level < 393.0 pg/mL have no difference in survival compared to those who have a higher level of activin A (≥ 393.0 pg/mL) (*p* > 0.05) (Figure 3 and Table 5).

**Table 5:** Chi‐square analysis of cut‐off value for activin A level (393 pg/mL) and 1‐year mortality outcome.

Activin A level cut‐off	Deceased (*n* = 44)	Survived (*n* = 102)	*p* value
< 393.0 pg/mL, *n* (%)	17 (38.6)	41 (40.2)	0.860
≥ 393.0 pg/mL, *n* (%)	27 (61.4)	61 (59.8)	

## 4. Discussion

In this study, the level of activin A in patients with ASD‐associated PAH did not associate with 1‐year mortality. There was no increased risk for mortality within 1 year for patients with higher activin A levels (≥ 393.0 pg/mL).

Recent studies have suggested the role of activin A as a valuable prognostic biomarker for PAH due to its high secretion level in patients with PAH [10–12]. Interestingly, this is not exclusive to idiopathic, heritable, or anorexigenic‐induced PAH, since our previous study also showed that activin A level was increased by 37.60% in CHD with PAH as compared to CHD without PH and 83.25% to control subjects (mean ± SD: 503.68 ± 179.69 pg/mL, 366.02 ± 68.56 pg/mL, and 274.85 ± 49.42 pg/mL, respectively) [8]. In this study, we confirmed that the level of serum activin A in ASD‐associated PAH was indeed high (527.1 ± 283.3 pg/mL), closely similar to our previous study [8]. However, the serum activin A level has no benefit in predicting 1‐year mortality among the subjects.

The French EFORT study reported that activin A values have a prognostic cut‐off value to predict PAH development. The increased level of activin A above 393 pg/mL was found in idiopathic, heritable, or anorexigenic‐induced PAH patients compared to healthy subjects. Furthermore, subjects with a low level of activin A (< 393 pg/mL) exhibited significantly lower percentages of death or lung transplantation compared to those with a high level of activin A in these subjects [6]. Meanwhile, the same cut‐off value does not apply in our study. There was no significant difference in terms of 1‐year mortality in our subjects between low and high levels of activin A, based on this cut‐off value. This could be due to the difference in the measurement method between the study by Guignabert et al. and this study [6], or the difference in the subject population characteristics. However, based on our analysis, the activin A level also offered limited predictive utility regarding 1‐year mortality outcome in our population as well.

Despite the same vascular changes and the same hemodynamic outcome, ASD‐associated PAH and other types of PAH have different pathophysiology that may affect the mortality outcome. A previous study also pointed out the difference in prognosis between CHD‐associated PAH and IPAH [13]. In PAH, the risk of mortality is closely related to right heart failure, which is driven by the increased pulmonary arterial pressure due to irreversible vascular remodeling in the pulmonary vasculature [14]. On the other hand, our study showed that in the deceased group, there were signs of worsening right heart function depicted by the higher RA area and mRAP, and a sign of heart failure showed by decreased TAPSE, higher NT‐proBNP level, and wasting syndrome. However, the deceased group also showed a 2‐fold increase in the proportion of subjects developing Eisenmenger syndrome compared to the subjects who survived. It gave a hint that most mortality cases in our subjects might not be solely due to heart failure, but due to the worsening right‐to‐left shunt as well.

Activin A is an isoform of activins, which are glycoproteins derived from the dimerization of inhibin family members that belong to the TGF‐*β* superfamily of growth and differentiation factors. Activation of activin A signaling is one of the major pathways in PAH development and progression [6]. In the lung, activin A was abundantly expressed in pulmonary artery endothelial cells and smooth muscle cells [6, 15]. The autocrine activity of endothelial cell–derived activin A induces degradation of BMPRII and, if activated, disrupts endothelial cell homeostasis [3, 10]. In pulmonary artery smooth muscle cells, upregulation of activin A provokes TGF‐*β* superfamily signaling imbalance, leading to activation of BMP inhibitors, gremlin, and noggin, which results in pulmonary vascular smooth muscle cell hyperproliferation [3, 11]. Recent studies have also confirmed the role of activin A in systemic vascular structure. Researchers found that activin A is involved in smooth muscle proliferation, enhances endothelial dysfunction through increased oxidative stress, and decreases endothelium‐dependent relaxation systems in essential and secondary hypertension [16, 17]. The same phenomenon was also observed in our study. Aside from the correlation between activin A and several right atrial parameters, activin A showed positive correlation with age and systolic blood pressure as well [17]. This further proved the importance of activin A in vascular homeostasis. Of note, due to the fact that the increase of pulmonary arterial pressure in ASD‐associated PAH was secondary to the left‐to‐right shunt, the change in activin A levels did not directly reflect the degree of severity of the vascular homeostasis in subjects with ASD‐associated PAH [17]. The relation between activin A and age, blood pressure, RA area, and RA pressure was modestly explained by the course of disease in ASD‐associated PAH, which involves vascular remodeling and changes in vascular cell function, resulting in increased RA pressure.

It is strongly believed that serum activin A could predict survival in idiopathic, heritable, or anorexigenic‐induced PAH and serves as independent for other known prognostic factors [6], as current Phase 2 and Phase 3 clinical trials of sotatercept, a first‐in‐class fusion protein constitute of human activin receptor type IIA extracellular domain fused to the Fc domain of human IgG1, showed improved survival in patients with PAH [12]. However, in the specific type of PAH, more specifically ASD‐associated PAH, the prognostic value and target treatment of the activin A pathway may be different; therefore, it warrants further study. Our data shows that in the baseline, serum activin A level did not differ based on the mortality data within a 1‐year follow‐up from the index of diagnosis. However, further research needs to be performed to clarify the result.

This study had limitations that need to be mentioned. Firstly, the selection of subjects was derived from a tertiary referral center which received patients with worse clinical conditions which affected the increase in outcome (mortality). Our hospital was the tertiary referral center for PH in the region; therefore, most PAH patients had the moderate to high‐risk stratification in the inclusion/baseline, and they were routinely followed up in our PH center. Secondly, the selection of 160 subjects, due to the availability of ELISA kits for activin A measurement, was not a large consecutive enrollment but a case control method (despite consecutive selection after group allocation). Last but not least, selection bias toward more severe clinical and hemodynamic phenotypes may have been present, as the subjects were partially defined based on all‐time mortality, with 50% of the 160 subjects dying beyond a 1‐year follow‐up period. This may have resulted in an overrepresentation of higher risk individuals and the inclusion of relatively severe cases within the control group. This factor may have influenced the magnitude of the observed associations and limited the generalizability of our findings to a broader, unselected population. Therefore, larger cohorts with more diverse clinical and hemodynamic characteristics are needed to better define prognostic associations.

## 5. Conclusion

In conclusion, serum activin A level, at the index of PAH diagnosis, increased and significantly correlated with age, systolic blood pressure, and right atrial pressure measured by echocardiography in ASD‐associated PAH. Serum activin A, at the index of PAH diagnosis, has no association with 1‐year mortality in patients with ASD‐associated PAH.

## Funding

This study was funded by the Directorate of Research Universitas Gadjah Mada (Direktorat Penelitian UGM) and Tim Peningkatan Reputasi UGM Menuju World Class University–Kantor Jaminan Mutu UGM under the scheme Post‐Doctoral Research Program (Number 3662/UN1.P.II/Dit‐Lit/PT.01.03/2023).

## Conflicts of Interest

The authors declare no conflicts of interest.

## Supporting information


**Supporting Information** Additional supporting information can be found online in the Supporting Information section. Table S1. Comparison of baseline characteristics between subjects (*n* = 160) and nonselected patients (*n* = 511) from the COHARD‐PH registry.

## Data Availability

The data that support the findings of this study are available from the corresponding author upon reasonable request.
