# Supplementary material for: Serum Activin A Level and 1‐Year Mortality in Atrial Septal Defect‐Associated Pulmonary Artery Hypertension: A Case‐Control Study From the COHARD‐PH Registry
Source: Int J Vasc Med. 2026 May 25;2026:7787559. doi: 10.1155/ijvm/7787559 (PMC13200696; doi:10.1155/ijvm/7787559)
Supplement: Supplementary file 1 — Supporting Information Additional supporting information can be found online in the Supporting Information section. Table S1. Comparison of baseline characteristics between subjects (n = 160) and nonselected patients (n = 511) from the COHARD‐PH registry. [file IJVM-2026-7787559-s001.docx]

Table 1. Comparison of baseline characteristics between subjects (n=160) and non-selected patients (n=511) from COHARD-PH registry

| **Characteristics** | **n** | **All registered patients**  **(n=671)** | **n** | **Selected patients**  **(n=160)** | | **n** | **Non-selected patients (n=511)** | **p-value*** | **p-value**** |  |
| --- | --- | --- | --- | --- | --- | --- | --- | --- | --- | --- |
| **Clinical data** | | | | | | | | | | |
| Age (years) | 671 | 36.74 ± 12.87 | 160 | 37.29 ± 13.48 | | 511 | 36.57 ± 12.68 | 0.921 | 0.686 |  |
| Sex females | 671 | 544 (81.07) | 160 | 130 (81.25) | | 511 | 414 (81.02) | 0.997 | 0.948 |  |
| 6MWD (m) | 623 | 247.6 ± 164.2 | 112 | 279.3 ± 98.69 | | 511 | 240.7 ± 174.7 | 0.798 | 0.500 |  |
| Systolic BP (mmHg) | 652 | 128.4 ± 35.71 | 141 | 133.8 ± 23.39 | | 511 | 126.9 ± 38.3 | 0.991 | 0.894 |  |
| Hemoglobin (g/dL) | 659 | 13.75 ± 2.84 | 148 | 12.47 ± 5.19 | | 511 | 13.46 ± 3.54 | 0.708 | 0.406 |  |
| Hematocrit (%) | 629 | 42.47 ± 6.18 | 128 | 43.38 ± 6.21 | | 501 | 42.24 ± 6.16 | 0.146 | 0.050 |  |
| Creatinine (g/dL) | 629 | 0.83 ± 0.28 | 128 | 0.9 ± 0.43 | | 501 | 0.82 ± 0.23 | 0.143 | 0.049 |  |
| NT-proBNP (pg/mL) | 550 | 1,906.0 ± 3,632.0 | 116 | 2,917.0 ± 3,909.0 | | 434 | 1,636.0 ± 3,509.0 | <0.0001 | <0.0001 |  |
| Eisenmenger syndrome | 653 | 67 (9.99) | 154 | 28 (17.5) | | 499 | 39 (7.63) | 0.001 | 0.0002 |  |
| WHO f.c | 622 |  | 140 |  | | 482 |  | 0.0036 | 0.0008 |  |
| I-II |  | 541 (87) |  | 110 (78.6) | |  | 431 (89.4) |  |  |  |
| III-IV |  | 81 (13) |  | 30 (29.4) | |  | 51 (10.6) |  |  |  |
| Pericardial effusion | 658 | 62 (9.4) | 152 | 15 (9.8) | | 506 | 47 (9.3) | 0.977 | 0.830 |  |
| **Right heart catheter** | | | | | | | | | | |
| mPAP (mmHg) | 651 | 44 ± 18.92 | 140 | | 48.95 ± 18.1 | 511 | 42.64 ± 18.93 | 0.0004 | <0.0001 |  |
| mRAP (mmHg) | 642 | 10.85 ± 5.5 | 139 | 10.45 ± 5.53 | | 502 | 10.98 ± 5.48 | 0.299 | 0.120 |  |
| PVRi (WU.m^2^) | 649 | 10.05 ± 12.64 | 138 | 14.41 ± 14.19 | | 511 | 8.87 ± 11.94 | <0.0001 | <0.0001 |  |
| Flow ratio | 650 | 2.56 ± 1.66 | 139 | 2.15 ± 1.43 | | 511 | 2.68 ± 1.71 | 0.0007 | 0.0001 |  |
| **Echocardiography** | | | | | | | | | | |
| RA area (cm^2^) | 664 | 38.81 ± 15.05 | 153 | 35.47 ± 15.03 | | 511 | 39.81 ± 14.93 | 0.0007 | 0.0001 |  |
| RA pressure (mmHg) | 662 | 9.83 ± 5.62 | 154 | 6.57 ± 4.45 | | 508 | 10.81 ± 5.57 | <0.0001 | <0.0001 |  |
| LVEF (%) | 667 | 69.26 ± 9.07 | 157 | 70.41 ± 8.4 | | 510 | 68.91 ± 9.24 | 0.432 | 0.195 |  |
| TAPSE (mm) | 666 | 24.18 ± 5.9 | 156 | 22.43 ± 5.84 | | 510 | 24.72 ± 5.82 | <0.0001 | <0.0001 |  |
| TVG (mmHg) | 665 | 64.67 ± 34.49 | 154 | 74.62 ± 32.87 | | 511 | 61.67 ± 34.44 | <0.0001 | <0.0001 |  |

Continuous data was presented in mean±SD; categorical data was presented in n (%)

* p-value from Kruskal-Wallis test compared three groups (all registered patients, selected patients and non-selected patients)

** p-value from Mann-Whitney test compared two groups (selected patients and non-selected patients)

SD: standard deviation; BMI: body mass index; BP: blood pressure; 6MWD: six-minute walking distance; NT-proBNP: N terminal pro B type natriuretic peptide; WHO f.c= World Health Organization functional class; RA: right atrium; TAPSE: tricuspid annular plane systolic excursion; TVG: tricuspid valve gradient; LVEF: left ventricle ejection fraction; mPAP: mean pulmonary artery pressure; mRAP: mean right atrial pressure; PVRi: pulmonary vascular resistance index; WU: Wood unit

|  |  |  |  |  |  |
| --- | --- | --- | --- | --- | --- |
|  |  |  |  |  |  |
|  |  |  |  |  |  |
|  |  |  |  |  |  |
|  |  |  |  |  |  |
|  |  |  |  |  |  |
|  |  |  |  |  |  |
|  |  |  |  |  |  |
|  |  |  |  |  |  |
|  |  |  |  |  |  |
|  |  |  |  |  |  |
|  |  |  |  |  |  |
|  |  |  |  |  |  |
|  |  |  |  |  |  |
|  |  |  |  |  |  |
|  |  |  |  |  |  |
|  |  |  |  |  |  |
|  |  |  |  |  |  |
|  |  |  |  |  |  |
|  |  |  |  |  |  |
|  |  |  |  |  |  |
|  |  |  |  |  |  |
|  |  |  |  |  |  |
|  |  |  |  |  |  |
|  |  |  |  |  |  |
|  |  |  |  |  |  |
